# The Price of Human Evolution: Cancer-Testis Antigens, the Decline in Male Fertility and the Increase in Cancer

**DOI:** 10.3390/ijms241411660

**Published:** 2023-07-19

**Authors:** Jekaterina Erenpreisa, Ninel Miriam Vainshelbaum, Marija Lazovska, Roberts Karklins, Kristine Salmina, Pawel Zayakin, Felikss Rumnieks, Inna Inashkina, Dace Pjanova, Juris Erenpreiss

**Affiliations:** 1 Latvian Biomedical Research and Study Centre, Ratsupites 1-1k, LV-1067 Riga, Latvia; ninela.vainselbauma@biomed.lu.lv (N.M.V.); salmina.kristine@gmail.com (K.S.); pawel@biomed.lu.lv (P.Z.); felikss.rumnieks@biomed.lu.lv (F.R.); inna@biomed.lu.lv (I.I.); dace@biomed.lu.lv (D.P.); 2 Molecular Genetics Scientific Laboratory, Riga Stradins University, Dzirciema 16, LV-1007 Riga, Latvia; marija.lazovska@inbox.lv (M.L.); robertskarklins10@gmail.com (R.K.); jerenpreiss@gmail.com (J.E.); 3 Clinic iVF-Riga, Zala 1, LV-1010 Riga, Latvia

**Keywords:** cancer-testis antigens, parthenogenetic, polyploid giant cancer cells, PGCCs, genome fragility, phylostratigraphic analysis, innate immunity placentation, endogenous retroviruses, sex determination, male infertility, endocrine disruption, environmental pollution

## Abstract

The increasing frequency of general and particularly male cancer coupled with the reduction in male fertility seen worldwide motivated us to seek a potential evolutionary link between these two phenomena, concerning the reproductive transcriptional modules observed in cancer and the expression of cancer-testis antigens (CTA). The phylostratigraphy analysis of the human genome allowed us to link the early evolutionary origin of cancer via the reproductive life cycles of the unicellulars and early multicellulars, potentially driving soma-germ transition, female meiosis, and the parthenogenesis of polyploid giant cancer cells (PGCCs), with the expansion of the CTA multi-families, very late during their evolution. CTA adaptation was aided by retrovirus domestication in the unstable genomes of mammals, for protecting male fertility in stress conditions, particularly that of humans, as compensation for the energy consumption of a large complex brain which also exploited retrotransposition. We found that the early and late evolutionary branches of human cancer are united by the immunity-proto-placental network, which evolved in the Cambrian and shares stress regulators with the finely-tuned sex determination system. We further propose that social stress and endocrine disruption caused by environmental pollution with organic materials, which alter sex determination in male foetuses and further spermatogenesis in adults, bias the development of PGCC-parthenogenetic cancer by default.

## 1. Introduction

The increasing worldwide risk of cancer, which is particularly high in European countries and the US (e.g., in the UK, it is currently estimated to be over 50% [1]) and currently rising in younger adults [2], is alarming. Furthermore, men are more likely to both develop and die from malignant tumours [3]. Male fertility was also shown to be continually decreasing over the last 80 years of monitoring worldwide [4]. Are both tendencies causally linked? Cancer-testis-associated (CTA) protein-coding genes (expressed nearly selectively in the normal testis), which originated with the development of mammals and expanded in placentals and hominids, turned out to be oncogenic drivers, responsible for the poor prognosis in cancer patients of many solid tumour types [5,6,7]. In this review, we attempt to analyse this fatal link’s evolutionary root and contemporary drive.

**Figure 1:**
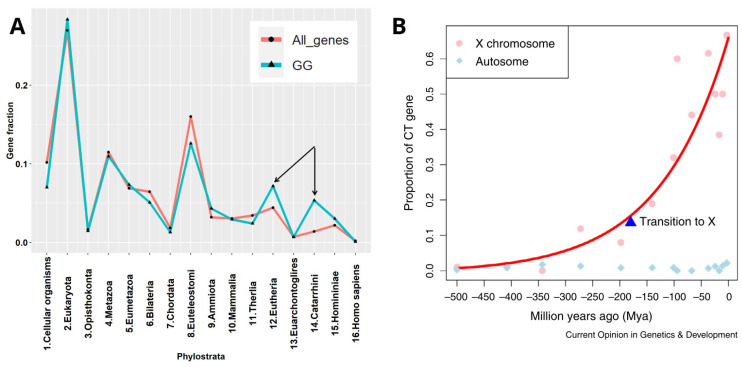
(**A**) The phylostratigraphic distribution of all available gametogenesis (GG) genes with phylostratigraphy information, with the distribution of all genes used as background. The two late splashes of CTA genes are marked by arrows. (**B**) Exponential transition from the autosomes and expansion of CTA genes to X- chromosome occurred ~170 Mya together with the origin of the X-chromosome, starting primate evolution. Adapted (**A**) from Ref. [8] under CC BY 4.0 licence, 2022 MDPI and reprinted (**B**) from Ref. [9], with permission from Zhang, Y.E. and Long, M, Curr. Opin. Genet. Dev.; published by Elsevier, 2014.

In our previous phylostratigraphy analysis of the human genome embracing the entire ~4-billion-year-long evolutionary timeline of life on Earth, by investigating the list of 1474 gametogenic (germ cell, meiotic, and CTA origin) genes, or GG, we revealed several peaks of evolutionary reproductive attractors [8] (Figure 1A). Beside the peaks in unicellulars (UC) (Strata 1 + 2) and early multicellulars (MC) (strata 4 + 5), and the peak in Stratum 8 (the Cambrian explosion of animal variety), we paid attention to two splashes of late GG that had evolved in Eutherians and Old World monkeys (strata 12–14). Those mainly included the CTA group of late origin [8]. With the origin of the X-chromosome, dated about 170 million years ago (Mya), the CTA genes which initially evolved in the mammalian autosomes, transited their exponentially expanding families onto the X-chromosome (Figure 1B). The evolution of X-linked CTAs was further hastened and is likely still going on in humans [9].

The following questions arise: (1) Why did these testis-associated genes evolve in hominids so late when sexual reproduction was already established? (2) And why are they associated with cancer? As the main X-located CT-MAGE genes code for antigenic proteins, this feature also needs an explanation of its link to fertility and cancer.

**Figure 2:**
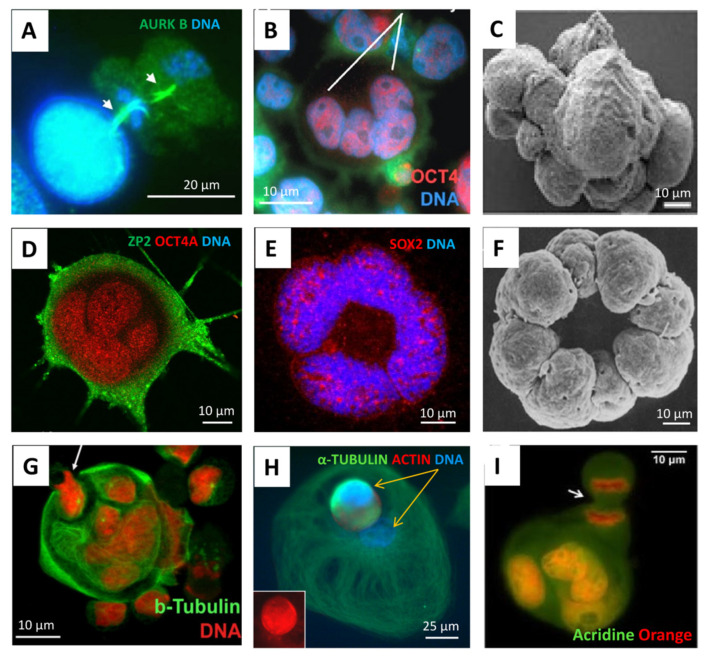
The similarity of PGCCs to early embryos, also showing asymmetric divisions and sporogenic-like processes; immunofluorescent staining or scanning EM in cell cultures: (**A**) The Namalwa Burkitt lymphoma cell line (44, X0), day 5 after 10Gy hit, treated with lactacystin 2 hrs before fixation, two subsequent midbodies of asymmetric reduction divisions are arrowed. (**B**) Lymphoblastoma WIL2NS (47, XY), 10 Gy—D5, the four-cell blastula-like PCGC is highlighted. (**C**) HEY ovarian carcinoma (82, XX) after paclitaxel treatment, mimicking cleavage blastula. (**D**,**E**) SK-MEL-28 melanoma (~90 XX,-Y) targeted treatment, D15. (**F**) Volvox 8-cell embryo, blastomeres united via cytoplasmic bridges. (**G**) Namalwa, 10 Gy—D13, the budding cellularised subnucleus is arrowed. (**H**) Breast cancer MDA-MB-231 (~64, XX) Doxorubicin—D13, the two different subnuclei of a giant amoeboid cell, one of which is spore-like, cellularised and mobile (actin/tubulin rich), are marked by yellow arrows. (**I**) Breast cancer MCF7 (69, XX), untreated, the budding cellularised subnucleus of a giant cell in the process of mitosis is arrowed. Reprinted (**A**,**B**) from Ref. [27], with permission from Erenpreisa, Je. et all., Oncoscience; published by Impact Journals LLC, 2015; (**C**) from Ref. [28], CC BY 4.0 licence; (**F**) from Ref. [40], with permission from Green, K.J. and Kirk, D.L., J. Cell Biol.; published by Rockefeller University Press, 1981; (**H**) from Ref. [20], CC BY 4.0 licence.

According to the popular evolutionary theory of cancer based on the phylostratigraphic data of cancer patient transcriptome databases, as well as that of driver oncogenes, the origin of cancer is dated very early, to the transition between UC and early MC organisms, (~2–1 billion years ago (Bya)) [10,11,12,13,14,15,16,17] creating in human cancers the rewired UC-MC gene regulatory network (GRN) [18], though it is considered a stepwise process [17,19]. Moreover, the genome rewiring in cancer, particularly in association with polyploidy and *c-myc*-related activation of bivalent developmental genes, was found to facilitate the coordinated expression of reproduction genes and proteins and favour the female meiotic pathway in solid TCGA (The Cancer Genome Atlas) tumours [8,16,20,21], which is potentially parthenogenetic, also in males [22]. Bruggeman et al. [23], who observed a massive expression of germ-cell-specific genes in cancer, came to the conclusion that it is a cancer hallmark; moreover, they showed, using TCGA lung adenocarcinoma as an example, that a higher germ cell gene expression signature was associated with poorer survival of the patients. All these data provide support for the old embryonal/parthenogenetic cancer theory [24,25,26]. In its current variant, the embryonal theory of cancer considers polyploid giant cancer cells (PGCCs) in somatic tumours to be exploiting a program of early embryogenesis [27,28] and sexually undetermined primordial germ cells [24,25,26]. For the historical arrow diagram describing advancements in cancer polyploidy research (including embryogenesis-like feature identification) from the 19th century onwards, see [29]. In the study of GG genes of 29 TCGA tumour types, we found that polyploidy enriches 17 tumour types with GG genes, while 10 of those tumour types have CTA/MAGE group members among their top 25 upregulated GG genes [8]. In further support, the microscopic observations from various laboratories during the two last decades have shown PGCCs, which are more numerous in genotoxically challenged cancers, to be similar to an early embryo (typically reaching the 8–16 cell (32C) stage), exhibiting meiotic and embryonal pluripotency markers [8,27,28,30,31,32,33,34] and being able to initiate tumours upon xenotransplantation of a single PGCC [35]. At the same time, PGCCs are capable of releasing their cellularised mobile offspring via asymmetric division, budding, or bursting. This process, termed ‘neosis’ [36], has the mixed features of multicellular embryogenesis and unicellular amoebal sporogenesis, and, not infrequently, literally recapitulates the latter in the process of developing drug resistance [12,37,38,39]. Some typical images of this process are compiled in Figure 2, with a Volvox 8-cell-bridged embryo image enclosed for comparison.

**Figure 3:**
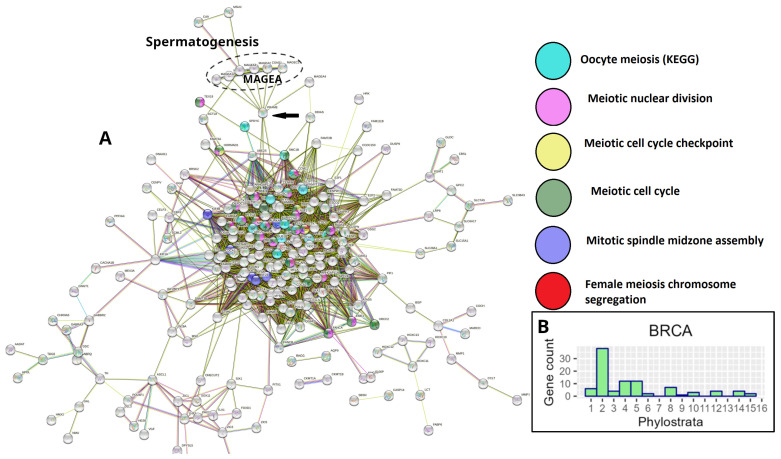
Gametogenesis profiles of TCGA breast carcinoma: (**A**) The STRING network of polyploidy-upregulated genes in TCGA breast carcinoma (BRCA), showing the presence of GG genes and enrichment of female-meiosis-related modules in the giant component with the MAGE/CTA subnetwork attached by the *PRAME* gene (arrow); (**B**) The phylostratigraphic distribution of ploidy-upregulated GG genes in TCGA-BRCA. Adapted (**A**,**B**) from Ref. [8], CC BY 4.0 licence.

However, a STRING PPI network analysis of gametogenetic (GG) genes in breast and many other common cancers from the TCGA database reveals that the giant component of the network, enriched for meiotic modules (including those of female meiosis/oogenesis), is bridged to the subnetwork of the CTA/MAGE group by the nuclear receptor transcriptional regulator PRAME antigen (Figure 3A). In Figure 3B, the GG phylostratigraphy distribution for polyploid BRCA samples, including expression of the evolutionarily late genes, is presented as a typical example for many common cancers [8].

Here, we tried to find the answers to the questions relating to CTA genes we formulated above, using data from the literature and also performing further in silico functional analyses of separate phylostratigraphy layers of the GG gene modules.

But first, we should start with a brief insight into the evolution of the human genome and CTA genes.

## 2. Evolution of the Human Genome via Segmental Duplications, Adaptation via Transposition and Fragility, CTA Origin, Reactivation of the X-Chromosome in Spermatogenesis, and X-Doubling in Male Cancer

Our human lineage started ~3.9 Bya with the origin of life through eubacteria, developed through multicellular eukaryotes, which underwent two rounds of whole-genome doubling at the base of vertebrate origin before the Cambrian explosion of the animal variety, at the second atmospheric oxygenation ~500 Mya; mammals originated ~250–200 Mya, primates ~90–57 Mya [19]. Compared to other mammals, the genomes of primates and particularly humans are enriched with large, interspersed segmental duplications (SDs), repeated in two or more genomic locations, with high levels of sequence identity. A strong association between SDs, genomic instability, and large-scale chromosomal rearrangements has been shown. The findings suggest that SDs have not only created novel primate gene families, but might have also influenced current human genetic and phenotypic variation on a previously unappreciated scale [41]. In total, 45% of the human genome is composed of transposable elements (TE): non-LTR (long terminal repeats) retrotransposons, short and long interspersed nuclear elements (SINE and LINE, correspondingly), LTR retrotransposons (endogenous retroviruses), and DNA transposons; TE are mostly epigenetically silenced [42]. Detailed analyses of the sequences of pairwise SD alignments have revealed that Alu, the most abundant RT class of mobile elements, is significantly enriched at the boundaries of SD pairs and restricted to younger subfamilies (AluY and AluS). The pairwise SD boundaries were shown to be fragile and the preferential sites of double-strand breakage. The fragile human genome sites assume a left-handed zigzag-like Z-DNA form of high energy tension and represent the sites of the high mutation and deletion rates [41,43]. Thus, SD and Alu’s repeats appear as the main origin of genome instability in primates and humans [41,44]. An updated analysis of the common fragile sites indicates their activation to be associated with replication stress and heterochromatin under-replication, which correlate well with chromosomal rearrangement and copy number variation and are likely causally linked to carcinogenesis [45,46]. Notably, the genome fragile sites are attractive for the meiotic recombination endonuclease SPO11 [47]. Vertically inherited endogenous ERV viruses possessing long terminal repeats (LTR) have also contributed to CTA evolution by producing tissue-specific variants (testis, brain, placenta), creating alternative gene promoters [48].

The evolution of CTAs in humans is tightly associated with the newest RT history, on the one hand, and the phylogenetic and ontogenetic history of the sex chromosome X, where the largest part of CTAs are located, on the other hand. The X-chromosome is enriched 2-fold for the autonomous RT LINE-1 (L1) that may also serve as DNA signals to propagate X-chromosome inactivation (through lncRNA) along the chromosome [49] while transposing via their smaller active fraction of the Alu-elements. The restricted subset of L-1 elements underwent an Eutherian burst [50] which could favour the Eutherian splash of CTA genes. Alu introduced the primate genomes to more than one million elements 60–35 Mya [50,51]. As shown in Figure 1B, CTA genes transited to and exponentially expanded on the X-chromosome. Given the high level of diversifying selection, it was suggested that CTA genes are primarily responsible for the observed rapid evolution of protein-coding genes on the X-chromosome [9] that involves the ongoing evolution of Alu repeats [52]. There are also the transcription-binding sites found within the Alu sequences, including the nuclear transcription factor family, in particular, steroid hormone receptors, progesterone and androgen receptors (PR and AR, correspondingly) [53]; they are associated with somatic sex-determination-regulating spermatogenesis, folliculogenesis, and placentation.

The early human embryo undergoes full genome activation at the 8-cell stage; the later-formed primordial germ cells (PGCs) maintain the paternally and maternally inherited imprinting patterns. This DNA methylation pattern is again rapidly erased when PGCs begin migrating towards the developing gonads and undergo reprogramming, starting the transition from mitotic to meiotic division during spermatogenesis [54]. Many CT genes located on the X-chromosome are involved in this reprogramming (see below). LINE-1 activation is essential for preimplantation development [55], they are expressed in round spermatids [56], and also the DNAse-hypersensitive nucleo-histone fraction of the mouse and human sperm is enriched in retrotransposon DNA [57,58]. The Xq26-28 fragile site, a region on the X-chromosome prone to breakage, has been linked to LINE-1 retrotransposition [59]. This region, particularly Xq27.3, includes the MAGE-A family of CTA genes, the youngest and the only transposing Ta subfamily of L1 amplified in the last 2 million years [60]. Non-autonomous Alu, which alters DNA methylation [61] and the autonomous, only human, transposing L1 subfamily, which generally favours methylation, is often clustered together in the fragile sites [62]. It remains to be mentioned that CTA genes are hyperactivated in cancers via demethylation [63].

From the above, it can be seen that the complex regulation of male CTA gene expression on the X-chromosome is RT-linked, associated with fragility, and also highly dependent on the DNA secondary structure and epigenetic modifications. The latter, as well as X-gene dosage, also depend on X-chromosome ontogeny, which is different for males (XY) and females (XX).

In the female karyotype, one of two X-chromosomes is inactivated (XCI). XCI in placental mammals is a dosage compensation mechanism that transcriptionally silences the majority of genes on one of the X-chromosomes in females. Because males have a single X-chromosome, this ensures dosage equivalence between males and females. Male cells reactivate their only X-chromosome during spermatogenesis [64]. Recent reports have shown that reactivation of the inactive X-chromosome, or a loss of the inactive and doubling of the active X-chromosome [65], a unique phenomenon that exists in many high-risk tumours in women, can transform the expression of many X-linked genes from monoallelic to biallelic. Therefore, Liu et al., (2018) [66] speculated that X-chromosome reactivation can inappropriately augment CTA expression in cancer. Therefore, it is highly interesting that our studies also revealed a high proportion of male tumour types with extra X-chromosome acquisition in the Mitelman tumour karyotype database, which thus presumably doubles the gene dosage of X-linked CTA genes [22]. Moreover, the studies on male breast cancers with an extra X-chromosome revealed the hypomethylation of the AR gene together with the CTA *MAGEA* family members, the coregulators of AR, both mapped on the X-chromosome’s q-arm [67]. The authors suggested that this cis-hypomethylation may lead to CTA and AR hyperactivation. Moreover, Talon et al. [64] proposed that genes encoded on the sex chromosomes act on autosomal genes to generate a differential regulatory and epigenetic landscape upon which later factors, such as hormones, act to counter or compound sex biases.

As suggested [68], and is generally accepted [69], CTAs emerged in evolution to protect male reproduction in mammals and particularly hominids (who possess large brains) from stress. The useful information on the functions of the most important CTAs/MAGE members related to stress is briefly compiled below.

## 3. The MAGE Protein Oncogene Family Functions in Gametogenesis and the Adaptive Stress Response

MAGEs (melanoma-associated genes, first found in melanoma) represent the most important group of CTA genes associated with cancer. *MAGE* genes are conserved in all eukaryotes and have expanded from a single gene in lower eukaryotes to ~40 genes in humans and mice. The type I MAGEs include the *MAGE-A*, *-B*, primate-specific-C, and mouse-specific Mage-a–like subfamily members. Type I MAGEs are called cancer-testis antigens (CTAs) because they are primarily expressed in the testis but are normally silent in other tissues; however, they are often aberrantly reactivated during oncogenic transformation and code for antigens recognized by cytotoxic T lymphocytes, and they are also involved in diseases other than cancer, including neurological disorders [70]. They are mostly located on the X-chromosome.

**Figure 4:**
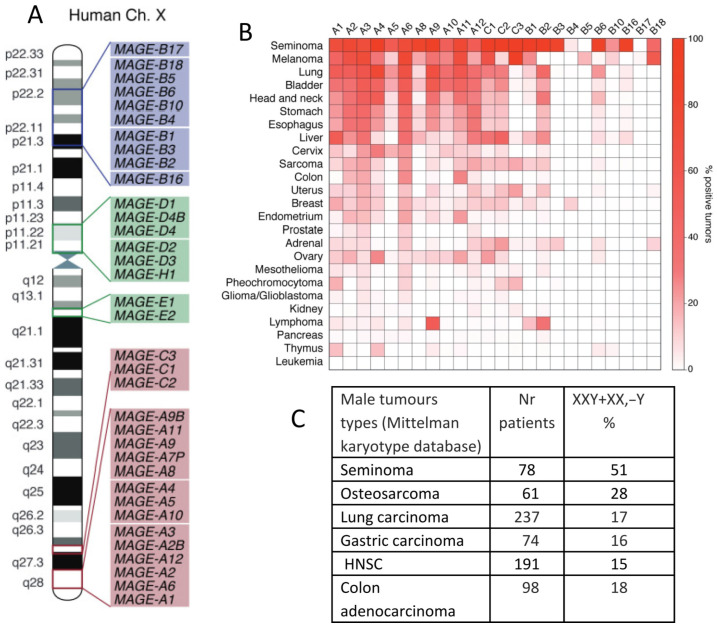
MAGE genes in tumours, their preferential location on the X-chromosome, and extra-X-chromosome gain in male tumours: (**A**) The locations of MAGE group genes on the human X-chromosome. (**B**) A heatmap of the expression of MAGE group genes in different cancer types. (**C**) A table of X-chromosome gain summary statistics in six cancer types from the Mitelman Database of Chromosome Aberrations and Fusions in Cancer. Reprinted (**A**,**B**) from Ref. [70], CC-BY licence; (**C**) from Ref. [22], CC-BY licence.

In contrast, the type II MAGEs, consisting of the *MAGE-D*, *-E*, *-F*, *-G*, *-H*, *-L*, and *NECDIN* genes, are more ubiquitously expressed in humans, particularly in the brain. They are typically not associated with human cancer and can be located on autosomes. In Figure 4A, the MAGE group I and II genes mapping on a human X-chromosome are shown. Notably, MAGE-A and -C subfamilies, most associated with cancer, are nested in the syntenic regions of the X-chromosome, the fragile locus Xq27.3 and locus Xq28, where the testis-associated genes are overrepresented. Some MAGE-B genes from the X-short arm are also involved in cancer. Figure 4B, also borrowed from the review [70], shows a heatmap of the percentage of various tumours that express each type of MAGE. It is interesting to compare it with the Table in Figure 4C, which showcases the male tumour karyotype cohorts showing the highest percentage of extra X-chromosome gain, presented by the same tumour types, with the upper row being seminoma in both figures.

It was supposed that the stress tolerance assigned by MAGEs might explain why many cancers capable of surviving anticancer treatments aberrantly express them [70,71]. At the molecular level, MAGEs are regulators of transcription factors, but many also bind to E3 RING ubiquitin ligases and, thus, regulate their substrate specificity, ligase activity, and subcellular localization. Moreover, a majority of CTA/MAGE are intrinsically disordered proteins (IDPs), toggling promiscuous links with various substrates, in a dosage-sensitive manner [72,73]. In general, the IDPs enable discrete cell transitions from one state to another and can change cell fate [73,74].

The MAGE-A group and MAGE-C1/C2 are involved in p53 suppression and cancer invasion [70,75]. In turn, the suppression of p53 can induce abnormal gametogenesis and parthenogenetic development in tumours [76]. MAGE (and CTAs in general) protect spermatogenesis under stress conditions (e.g., famine) in vivo [71].

CTA genes, including MAGEs, have been recently excellently reviewed [6,7,70]. Therefore, we only briefly indicate some functions of selected CTA genes which provide key points for our further analysis of GG STRING PPI networks.

The population of GAGE-expressing male and female germ cells is partially OCT4-positive [77].

*MAGE-A3/6* downregulates autophagy and apoptosis in response to cellular starvation [78] and also supports genome stability via the degradation of retrotransposon RNA [79].

- *MAGE-A11* forms complexes with *MAGE A3/6* and regulates AR function in spermatogenesis and somatic sex determination [80], and also interacts with AR and PR to favour embryo implantation [70].
- *MAGE-B2* (locus Xp21.2) coincides with the position of *NORB1*, whose doubling or deletion leads to dosage-sensitive sex reversal [81].
- *MAGE-C1/C2*—involved in p53 suppression and cancer invasion [70].
- *SPANX-N* and (A–D) (Xq261-27.3)—(A–D) is the human gene family derived from the rodent *SPANX-N*, they are responsible for sperm motility (*SPANX-N*) and sperm head packaging (A–D) [77,82], and were also found to be controlled by the nuclear lamina in melanoma [83].
- The antigenic *PRAME* is a very important master gene connecting the meiotic giant nucleus of the cancer cell genome network with the MAGE-A cluster (Figure 3A). As a nuclear receptor transcriptional regulator, it activates the embryonal stemness (through *OCT4A*) and PGC program (through *SOX17*) [84] and it also downregulates cell differentiation as a dominant retinoic acid receptor signalling gene [85]. So, PRAME can be crucial for cell fate change and soma-germ transition. PRAME is overexpressed not only in many solid tumours but also in myeloid leukaemia [86].

More than 300 human-specific genes and 1000 primate-specific novel genes appear to be implicated in brain development and male reproduction [87] and often share the chromosome location and functions. A large MAGE-A group of spermatogenic genes is located in the X-fragility Xq27.3 locus, which is associated with the FMR1-mental disability syndrome [88] coupled with a low sperm count in men [89] and mild ovarian failure in affected women [90]. Fragile X-Associated Tremor/Ataxia Syndrome (FXTAS) [91] is another pathology involving this site. A more detailed CTA-related brain-associated pathology review and discussion are out of the scope of this article.

These literature insights will help in interpreting the gametogenic gene STRING networks which we further analysed. Short synopsis on CTA genes is given in Box 1.

Box 1 **CTA genes evolution and function in short.**

CTA genes emerged in mammals for strengthening male gametogenesis in stress conditions and are almost selectively expressed in the testis (and also in the ovarium, placenta, and brain). The most important MAGEA group is located at the fragile site on the X-chromosome. CTA are under strong epigenetic control. When demethylated and abnormally overexpressed, they act as oncogenes, due to their effect on the tumour suppressor p53 and other mechanisms, while also activating embryonal stemness, primordial germ cells, and oogenesis. Their overexpressed proteins also acquire the properties of antigens.

## 4. STRING Network Analysis of GG Genes in the Human Genome along the Evolutionary Phylostratigraphic Axis

The list of 1474 gametogenic (GG) genes is compiled from the CTDatabase [92] cancer-testis genes, the germ-cell-specific genes from the work of Bruggeman et al. [23], and the MeiosisOnline meiotic gene database extended with a manually curated gene list (*SYCP1*, *SYCP2*, *SYCP3*, *SYCE1*, *SYCE2*, *HORMAD2*, *MAEL*, *MEIKIN*, *MEIOB*, *MEIOC*, *SYC E1L*, *TEX11*, *MAJIN*, *FAM9C*, *FAM9B*, *FAM9A*, *REC114*, *TEX19*, *BRME1*, *TEX14*, *MSH4*, and *TEX15*). For the purposes of this work, we have extended it further, adding genes of early embryogenesis (*POU5F1*, *ZP4*) and genes from the pregnancy/placentation functional modules recently identified by us to be upregulated in MDA-MB-231 cells on day 5 after doxorubicin treatment [20]. The phylostratigraphic distribution of these genes was determined using the gene phylostratigraphy data from the work of Trigos et al. [11]. This gene list, now designated GG+, is presented in Table S1 alongside their respective phylostratigraphic groups (phylostrata). The STRING PPI networks were constructed separately for phylostrata 4–5, 8, and 10–16 at medium confidence, while the phylostrata 1–2 network is republished [8]. The STRING database’s [93] web interface was used to prototype the networks and identify their giant components, after which the STRING network tables were downloaded and the final design of the networks was constructed using ggraph [94] and ggplot2 [95] in R.

**Figure 5:**
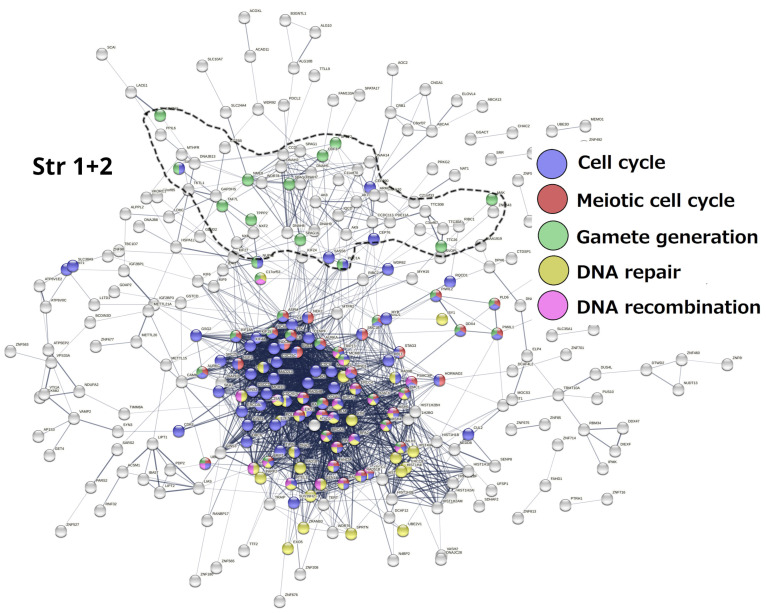
The STRING networks of GG genes correspond to the 1st and 2nd evolutionary phylostrata. Genes (nodes) belonging to enriched functional modules of interest are displayed in the form of pie charts. Adapted from Ref. [8], CC BY 4.0 licence.

Stratum 1 + 2 (Figure 5). The modules of the cell cycle, meiotic cell cycle, DNA repair and recombination, and gamete generation were revealed. It indicated the already-established emergence of meiosis and sex in UC eukaryotes. Notably, a loose subnetwork of gamete generation is not integrated into the dense network core component, including meiotic recombination and DNA repair genes. It may mean that, in this case, meiosis could be present in one of its evolutionarily earlier forms—endomitotic or zygotic [96,97,98].

**Figure 6:**
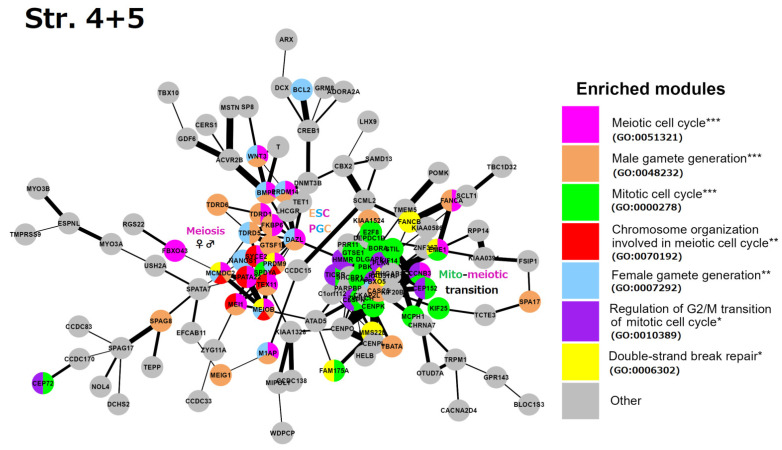
The STRING network of GG+ genes (Table S1) corresponds to the 4th and 5th evolutionary phylostrata. Genes (nodes) belonging to enriched functional modules of interest are displayed in the form of pie charts. The main enriched functional modules are also designated by the coloured text font. Hypergeometric test * *p*-value < 0.05; ** *p*-value < 0.01; *** *p*-value < 0.001.

Stratum 4 + 5 (Figure 6): Two interacting network clusters are presented. A central subnetwork is a cluster of meiosis networked with the generation of both gametes (gametic meiosis). The meiotic cluster (including PRDM9, MRE11, TEX11, MCMDC2, and other essential genes for the homologous pairing and programmed repair of DNA DSBs, like MCMDC2 [99]. This cluster also includes NANOS1 (light-blue), which downregulates mitosis during female germline development [100] and possesses the features of oncofetal oncogenes [101], with a direct link to DAZL. DAZL (triple-coloured) is a hub of early embryogenesis (ESCs) and the development of primordial bisexual germ cells (PGCs) [102]. Furthermore, three other key genes of the ESC and PGC determination, PRDM14, BMP4, and WNT3, are present [103]. Another big cluster highlights the mito-meiotic cell cycle transition. The genes of this cluster indicate replication stress (ATAD5), DNA replication and S/M and G2/M checkpoints (TICRR, CLSPN), DNA double-strand break repair via homologous recombination (FAM175A, RAD51AP1), delay in G2/M phase progression (GTSE1), inactivation of the anaphase-promoting complex, metaphase–anaphase transition in meiosis I (CCNB3), microtubule motor (KIF14), and remodelling of MTOC during oocyte maturation (FBXO5, CEP152). The cluster also includes the meiotic crossover junction endonuclease EME1.

In summary, we see the role of Strata 4 + 5 in the mito-meiotic transition induced via replication stress and DNA double-strand breaks (likely transiting the mitotic G2/M with its DNA damage checkpoint into meiotic prophase [104,105]), gametogenesis networked with meiosis (gametic meiosis), converging on the establishment of the Metazoan preimplantation embryo and germline lineage (PGC), and oocyte maturation with the oncofetal potential, which is also supported by the literature data and linking Stratum 5 with oncogenic driver genes [10,106]. 

**Figure 7:**
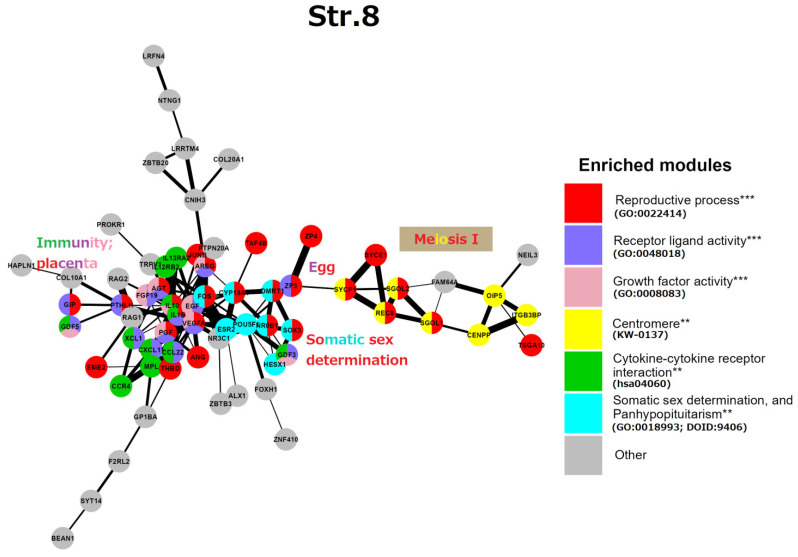
The STRING network of GG+ genes (Table S1) corresponds to the 8th evolutionary phylostratum of the human genome. Genes (nodes) belonging to enriched functional modules of interest are displayed in the form of pie charts. The main functional modules are also designated by the coloured text (e.g, red—reproductive process, yellow—centromere, etc.). Hypergeometric test ** *p*-value < 0.01; *** *p*-value < 0.001.

Stratum 8 (Figure 7): This GRN part embraces mostly “reproductive processes” (GO:0022414) at the level of individual organisms. A gene central to the network, FOS, represented by its AP-1 dimer (with JUNB), highlights a general stress response. In the reproductive context, FOS is critical for the upregulated expression of key ovulatory genes in human granulosa cells, mediated through hormonal receptor PGR and EGF signalling [107]. At the same time, FOS and JUN B are members of the “female pregnancy” GO module (GO:0007565). Thus, here the master stress-response protein FOS unites two somatic modules of reproductive processes: the cluster of endocrine somatic sex determination, on the right, and the immunity/placental cluster, on the left. Most genes of the GO “female pregnancy” module (GO:0007565), IL-1β, VEGFA, THBD, AREG, PGF, PTHLH, AGT, FOS, and JUNB, are interlaced with the immunity network of cell communication and angiogenesis knotted via cytokines IL-10 and IL-1β. In addition, we find a cluster related to conventional meiosis I including the centromeric cohesin REC8 and its stabilisers (SGO1,2) and the central elements of SC (SYCP1, SYCE1). This subnetwork is connected to the ZP3/ZP4 *zona pellucida* proteins (the vertebrate egg-coating glycoprotein interacting with ECM) that enclose the matured oocyte and early embryo [108]. Another link between the somatic regulation of reproduction to generative embryonic stem cells is seen through the inclusion of the POU5F1, a key to embryonic pluripotency and PGC development [109].

Here, the cluster of somatic sex determination (“the determination of sex and sexual phenotypes in an organism’s soma and involving endocrine regulation”) reveals key genes. DMRT1 (the double-sex-related transcription factor) is involved in sex determination and gonadal development (stimulation of Sertoli cells and ovarian follicles [110,111]). Its expression in PGCs is not sexually distinctive; moreover, DMRT1 is haploinsufficient for testicular development, and it can cause male-to-female sex reversal in the embryo. In humans, DMRT1 is critically required for the development of the testis during the foetal period. In the adult testis, DMRT1 is predominantly expressed in Sertoli cells and is also required in spermatogonia, enabling the restoration of their pool after sperm depletion. Another gene involved in epigenetic sex determination, CYP19A1 is involved in the androgen-to-oestrogen receptor conversion (AR-ER) by aromatase P450. Its activity in males is restricted by methylation, while the haploinsufficient-for-males DMRT1 is more methylated in females [112], both thus epigenetically preventing male-female sex reversal in the embryo. The actions of steroid androgens such as testosterone and dihydrotestosterone are mediated via the X-linked androgen receptor (AR). It becomes hyperactive (undermethylated) in breast- (regardless of patient sex) [113,114] and castration-resistant prostate cancers [115]. AR is also involved in the brain and some other tissue functions [116]. The third gene of this cluster NROB1 (DAX1) is related to sex determination and reversal through its link with glucocorticoid receptors (adrenal gland), while SOX3 is involved in sex determination and brain development. The balanced sex determination via this system provides the normal male/female birth sex index ratio (approximately 1). More about the hypothalamus-gonadal endocrine sex regulation can be found below, in the section devoted to male infertility. Short synopsis on the String analysis of strata 1–8 is presented in Box 2.

Box 2 **Short synopsis on the analysis of reproduction modules in evolutionary Strata 1–8.**

The in silico phylostratigraphic analysis of nearly 1500 genes known to be involved in reproduction revealed the preservation and interconnection in the human genome of reproductive functional modules (gene networks) that had emerged throughout the whole evolution of life on Earth. In unicellulars (Strata 1 + 2), meiosis and sexual reproduction were already established. In early multicellulars (Strata 4 + 5), the mechanism of soma to germline transition emerged with a link to meiosis, ESC, and embryo development (important for parthenogenetic cancer initiation). In complex animals (Stratum 8), the immune response and proto-placentation (providing invasion and vascularisation) emerged simultaneously with somatic (hormonal) sex determination. Importantly, both are induced and linked by early stress response genes and involve the master embryonal stemness gene OCT4 (*POU5F1*), with male sex determination being more genetically and epigenetically restricted than female.

The aromatase CYP19 for the AR-ER transition coded by its respective sex determination gene is expressed in the reproductive organs and the brain of most mammals. Notably, in primates, an LTR-ERV-promoted transcriptional variant of this gene confers the alternative expression to the placenta [117].

In summary, we see the organism-level regulation of reproduction via cytokine-cytokine receptors and growth factors for cell communication in immunity fused with the placental regulators, mostly for angiogenesis linked by the general early stress response gene FOS to the establishment of the vertebrate endocrine somatic sex determination. This somatic regulation also has links to the established meiosis and the mature oocyte-early embryo vertebrate development. As it looks, the whole set, if not fine-tuned for males or altered, is inclined by default for embryo sex reversal and female germline development.

The placenta module, standing out in Phylostratum 8, much before the development of Eutherians, seems confusing. Therefore, in the next section, we inserted a mini-review devoted to placenta evolution and its cancer-related issues.

## 5. Mammalian Placenta Evolution, Immunity, and the Enrichment of the “Female Pregnancy” GO Module in Genotoxically Challenged PGCCs

**Figure 8:**
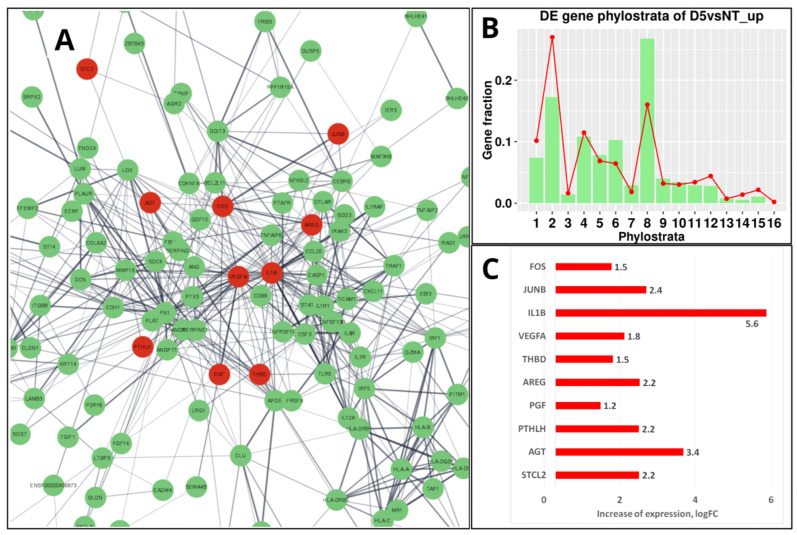
The transcriptome response of MDA-MB-231 cells to 100 nM-24 h Doxorubicin (DOX) treatment sampled on day 5: (**A**) The STRING network of upregulated genes (green-marked) in the 8th phylostratum; ten of them belonging to the ‘female pregnancy’ module (GO: 0007565) are coloured red and occupy central positions in the network. (**B**) The DOX-upregulated gene phylostratigraphic distribution (green bars against the red-lined whole-genome reference) showing the strong activation of Str 8 via DOX. (**C**) The “female pregnancy module” upregulated genes (log-folds). (**B**) Republished from Ref. [20], CC BY 4.0 licence.

Before mammalians, in the teleosts (Teleostomi, Phylostratum 8), a “follicular placenta”, lined with microvilli and surrounded by the highly vascularized tissue to facilitate maternal-foetal exchange, has arisen several times, making it a model for the evolution of placentation [118]. However, it is only in mammals that the placenta has developed from a trophoblast lineage specified in an embryo from the morula stage [119], which in humans is represented by its invasive variant [120,121]. It is noteworthy that the resulting placental structures of mammals and their associated trophoblast cell populations appear not to be governed by particular master genes but rather depend on widely expressed transcription factors embedded into the intercellular communication network of immunity cytokines and growth factors (which also evolved in Euteleostomi [11]) and operating in a combinatorial manner [120]. There are few predominantly placenta-specific genes, e.g., *GCM1*, a transcription factor that plays a role in controlling the formation of syncytial trophoblast (STB) [122]. Intriguingly, it was found recently that ancestral retroviral infections have provided a source of novel protein-coding genes that have played a role in the Eutherian evolution. In many species, the placenta expresses a range of endogenous retroviruses (ERVs) that are involved by integrating part of their DNA (*env*) into the regulatory part of placental genes to produce the cell-fusing syncytins of the syncytiotrophoblast, the most specific structural component of the placenta [120,123,124,125]. The transmembrane fraction of syncytins called the immunosuppressive domain (ISD), which can induce the severe immunosuppression of host cells, is therefore potentially oncogenic. The proviral activity of the retrogenes is controlled by the innate immune response to viral and cytosolic DNA fragments (the cGAS-STING pathway [126]). However, in senescent and cancer cells, this pathway may be unleashed, particularly by anticancer treatment, resulting in the activation of the GO module of ‘female pregnancy’ in the PGCCs undergoing repeated rounds of mitotic slippage [20]. As reported, the induced change of gene networks in the Doxorubicin-treated triple-negative breast cancer MDA-MB-231 cells (exampled and illustrated in Figure 8A at day 5 post-treatment) is mostly highlighted by the differentially expressed gene phylostratigraphic distributions markedly peaking at the 8th phylostratum (Figure 8B), which is related to innate immunity and hubbed, in particular, by IL-1β [20]. The highly upregulated IL-1β shares the enriched ‘female pregnancy’ module (Figure 8A,C) with modules related to innate immunity, which has originated in Euteleostomi (see above in Figure 7).

## 6. String Network Analysis (Continuation) Strata 10–16 

**Figure 9:**
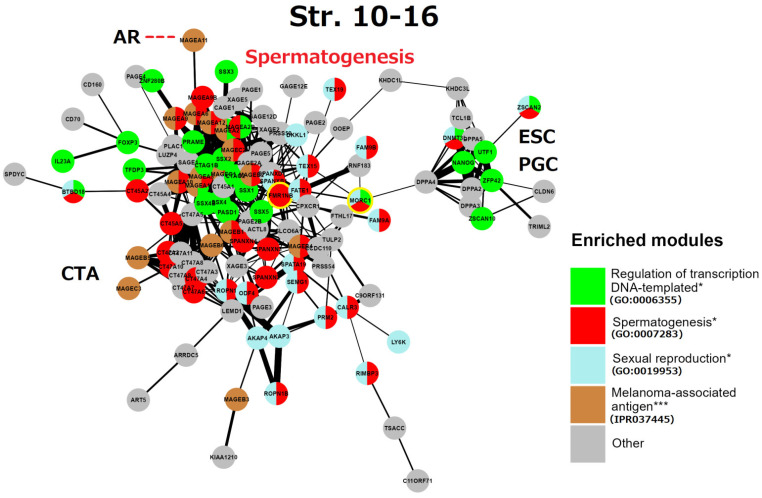
The STRING network of GG+ genes (Table S1) corresponds to the 10th-16th evolutionary phylostrata. Genes (nodes) belonging to enriched functional modules of interest are displayed in the form of pie charts. The giant component of the network belongs mostly to MAGE-associated genes of spermatogenesis, it is connected to the subnetwork of the embryonic stem cell (ESC) and primordial germ cell (PGC) development. The link of MAGE-A11 to the androgen receptor (AR) is indicated by a red dashed line. The two genes sharing spermatogenic functions with brain development are yellow-marked. Hypergeometric test * *p*-value < 0.05; *** *p*-value < 0.001.

This gametogenic subnetwork of the human genome in Strata 10–16 (Figure 9) is of late evolutionary origin (mammalian-human). It is mostly represented by X-linked CTA protein interactions highlighting spermatogenesis. It is enriched with the MAGE group and related reproduction processes. In particular, all these genes are also potential oncogenes [5,6,7]. The densely intertwined CTA-MAGE core of the network (predominantly from the long arm Xq27-28, (see details in Figure 4)) drives spermatogenesis from the proliferation of spermatogonial cells, including the FATE1 (Xq28) gene. This important gene, which is strongly active in embryonic and adult spermatogenesis, is a key factor in decreasing the sensing of stress [127] and is harnessed by cancer cells to escape apoptotic death and resist the action of chemotherapeutic drugs [127,128].

Two other notable genes are TEX15 (8p12) required for DNA DSB, chromosome synapsis, and meiotic recombination in spermatocytes; while SPANXN and (A-D) families (Xq26.2 -27.3) are strictly associated with spermiogenesis (sperm motility and head packaging [129]) and are strongly involved in melanoma genesis [130]. Furthermore, the gene FMR1NB (Xq27.3) has both spermatogenic [131] and oncogenic functions [132]. It is the closest neighbour of the gene FMR1 (associated with mental disability and reduced fertility X-fragility syndrome). The aligned looser subnetwork of CTA genes includes the SPANX-N family and the MAGE B1-B6 group (neighbours of the NROB1 gene of dosage-sensitive sex determination [81] on the short arm of the X-chromosome (for localisation details see Figure 3). The CTA45A gene family is composed of the testis-restricted cancer genes, whose tumorigenic, invasive (EMT-promoting) capacity is enhanced by growth factors as examined in breast cancer. The genes of the CTA47A family, which are testis-restricted, form a compact group on the Xq24 locus and also interact with CTA genes on the Xq28 (MAGEA1) and Xp22.2 locus [133]. The function of the CTA 44-47 families is evidently spermatogenesis-oncogene-related (as seen in GeneCards) [134] (as manually labelled).

A more remote subnetwork, including the DPPA group—development pluripotency-associated genes—belongs to the ESC and PGC module, it importantly includes the ESC master gene NANOG, which is strongly expressed in foetal gonocytes and in situ germ cell cancer [133]. This subnetwork also includes DNMT3L, inherited by maternal imprinting which promotes neural tube, placenta, and ovary development, and inactivates RT in the male germline, safeguarding it from mutations. This ESC/PGC subnetwork is converging to DPPA4—the development pluripotency chromatin modifier, which links it (through the multidrug-resistance gene MDR1 for the brain–blood barrier) to the FATE1-mediator gene bridge connecting large clusters of spermatogenesis and CTA/MAGE-A. In turn, the CTA/MAGE-A subnetwork converges to the PRAME gene. The PRAME gene (chr #22) represses endoderm differentiation, activates the *POU5F1* promoter for the induction of ESC and modulates *SOX17* to function as a master PGC gene [84]. In addition, PRAME is activated by IFN-γ closely associated with cGAS-STING (sensing cytosolic DNA) and IL1β signalling [135].

In summary, the reproductive CTA genes of the Strata 10–16, which originated in mammals and expanded in primate evolution and, even more, in humans, are largely X-linked or maternally imprinted, and aimed in general for the support of the male germline development, from spermatogonia to sperm maturation. They provide stress protection for the male reproductive system; however, paradoxically, they also acquired the functions of antigens and oncogenes. The CTA module of this stratum is capable of activating the modules of ESCs and sexually undifferentiated PGCs, due to the included ESC master gene *NANOG*. Activated *NANOG*, in turn, is well-known for its cooperation with the pluripotency transcription factors *POU5F1* (*OCT4*) and *SOX2*, which are the “pioneers” of development [136]. Short synopsis on the analysis of reproduction modules in evolutionary Strata 10–16 (Box 3). It seems important to further clarify the potential carcinogenic link between late spermatogenetic Phylostrata of mammals and humans and the Phylostratum 8, which evolved much earlier.

Box 3 **Short synopsis on the analysis of reproduction modules in evolutionary Strata 10–16.**

The phylostratigraphic in silico analysis of gametogenetic genes reveals the dominating spermatogenesis regulation employed by multiple families of X-linked CTA cancer-testis associated genes with the connection to the early embryogenesis and primordial germ cell development-driving master gene *NANOG*. CTA genes have a path to somatic sex determination through the androgen receptor (AR), while the whole CTA group is linked by its master regulator PRAME to meiosis. This connection is common for many cancers, also female, as shown in Figure 4.

## 7. The Causal Link between the GG Genes of the 8th Phylogenetic Stratum and CTA-Enriched Strata 12 and 14 in Spermatogenesis and in Cancer Progression

**Figure 10:**
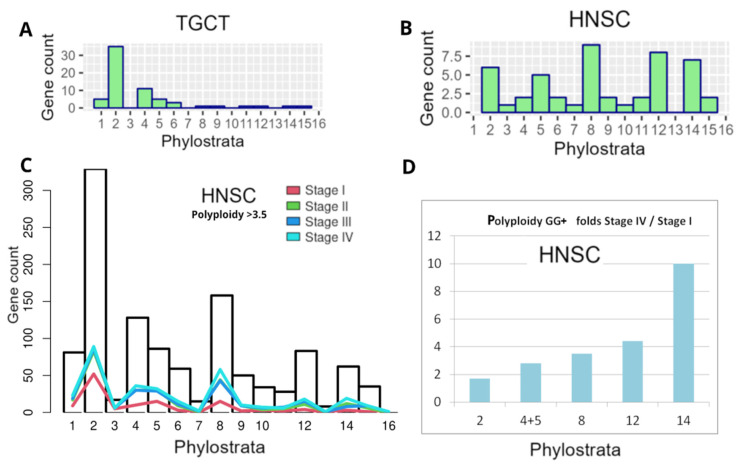
Phylostratigraphy analysis of GG+ genes differentially expressed in polyploid cells of two tumour types and depending on tumour stage, from TCGA database: (**A**,**B**) differential expression of GG between diploid and polyploid tumours (using Quinton et al.’s data [137] in TGCT and HNSC); (**C**,**D**) HNSC: (**C**) the gene counts (from GG+ Table S1 presented as bars per Phylostrata) in polyploid samples (threshold > 3.5) compared with normal tissue, for I-IV cancer stages; (**D**) the fold increase in GG+ gene count per Phylostrata from Stage I to Stage IV. (**A**,**B**) Republished from [8] under Creative Commons CC-BY licence.

In our previous bioinformatic study on the distribution of polyploidy (WGD)-upregulated GG genes in TCGA tumours (using Quinton et al.’s data [137] on the differential expression between diploid and polyploid tumours), we characterised the GG gene distribution histograms of 17 primary tumour types from the TCGA database [8]. The typical phylostratigraphy profile for common solid cancers with the dominating Stratum 2 is presented for polyploid BRCA in Figure 4B. Between 17 tumour types selected via polyploidy, there were two distinguished and opposite patterns for testicular germ cell tumour (TGCT) and head and neck squamous carcinoma (HNSC). In polyploid TGCC, Stratum 8 and further strata were absent, while in HNSC, on the contrary, those were highly expressed (Figure 10A,B).

To understand the situation with testicular germ cell cancer (TGCT), it is useful to learn about its development in ontogenesis. The TGCT are derived in adulthood from dormant PGCs [138] and PGC/gonocyte-like germ cell neoplasia in situ (GCNIS) [139]. Accordingly, they do not undergo the full spermatogenic pathway and it is reported that some of them do not express CT antigens [140]. From our data, it follows that regulation of spermatogenesis encoded by CTAs in mammals needs the expression of the proteins encoded in Stratum 8 (immunity and sex determination) and that polyploidy-related testicular tumours are likely void of the regulation via both sex determination and CTA.

Here, for HNSC strongly expressing Str. 8 and CTA genes associated with polyploidy (see Figure 3B), we decided to analyse the distribution of the differentially expressed (compared to 44 normal samples) GG+ in the phylostratum peaks depending on the cancer stage and polyploidy, as verified with a large number of TCGA-HNSC samples (Stage I–25, Stage II–74, Stage III–74, and Stage IV—259 samples). Polyploidy samples were filtered with a threshold > 3.5 using GG+ (Table S1). As can be seen in Figure 10C, the number of the GG genes available for analysis, which is generally the highest in Str 2, also undergoes predominant peaking there at Stage I, along with the increase in Str 4–5 and 8, whose GG numbers are relatively smaller, while the reaction of Str 12 is weak, and of Str 14 practically absent. However, with cancer progression and particularly at Stage IV of disseminated metastatic cancer, the profile is changing its vector, favouring a more significant GG+ contribution of Str 8 and later strata. The difference in fold increase in the involved GG+ gene count in each stratum of interest comparing Stage IV with Stage I, as presented in Figure 10D, clearly shows this tendency and the particularly big response of the hominid CTA MAGE-rich Str.14 (10-fold). It indicates the crucial role of CTA genes in the polyploidy-related metastases of this cancer type. Although HNSC has a good prognosis for patient survival at the initial stages, recurrent or metastatic HNSC is largely incurable [6].

The genome instability of mammals and endocrine disruption enhanced by the current environmental pollution and climate change synergistically potentiate male infertility, embryonic sex reversal probability, and increase cancer risk. These factors are also linked to developmental disorder risk. The following sections will briefly outline these aspects.

## 8. The Global Decline of Male Fertility, the Link to Cancer Risk, and the Consequence of Endocrine Disruption

**Figure 11:**
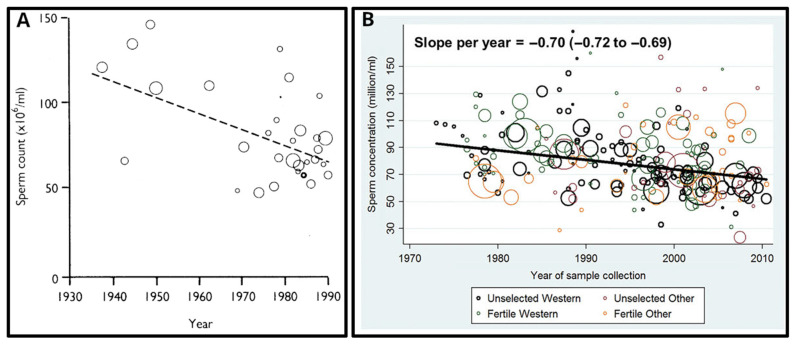
The decline of male fertility in the 80-year period (1930–2010): (**A**) Linear regression of mean sperm concentration reported in 61 countries (represented by circles whose area is proportional to the logarithm of the number of subjects in the study) each weighted according to number of subjects, 1938–1990. (**B**) Significant decline of sperm concentration in Western and other countries (slope per year −0.70 million/mL; 95% CI: −0.72 to −0.69; *p* < 0.001) over the study period when using simple linear models. Reprinted (**A**) from Ref. [141], with permission from Carlsen, E. et al., BMJ; published by BMJ, 1992 and (**B**) from Ref. [142], with permission from Levine, H. et all, Hum. Reprod.; published by Oxford University Press, 2017.

The first evidence of a global impairment of male reproductive health was published in 1992 [141]. The authors reported a significant (two-fold!) decrease in mean sperm counts from 113 million/mL in 1940 to 66 million/mL in 1990 in the United States, and many European, South American, African, and Middle-East countries (Figure 11A). They also reported the concomitant increase in the pathologies and morbidities of the male reproductive tract such as testicular cancer, cryptorchidism (undescended testicles in newborns), and hypospadias.

Many researchers considered the data from this publication as a kind of slowly ticking bomb threatening mankind, and many studies were initiated to either confirm or overturn these data. The following publications were very controversial—some of the studies did not confirm the decline in semen quality over time [143,144], whilst the data from the others supported such a decline [145,146,147,148]. However, there were important limitations connected to all these studies: (1) the data were poor or highly variable; (2) the validity of the statistical methods was questionable; (3) different study populations were investigated; and (4) confounding factors such as age and abstinence time (time between sample collection and last ejaculation) were not taken into account in all studies [142].

The first systematic review and meta-regression analysis of temporal trends in sperm counts was published in 2017 [142]. It reported a significant overall decline in semen quality in men from North America, Europe, Australia, and New Zealand, analysed between 1973 and 2011. Declines were most pronounced among men unselected through fertility—they showed a decrease of 59% (−1.6% per year) over the study period (Figure 11B). Declining slopes remained unchanged after controlling for multiple covariates: age, abstinence time, method of semen collection, method of counting sperm, selection of population and study exclusion criteria, number of samples per man, and completeness of data. Thus, these data provided a robust indication of a decline in male reproductive health in North America, Europe, Australia, and New Zealand over the four decades.

**Figure 12:**
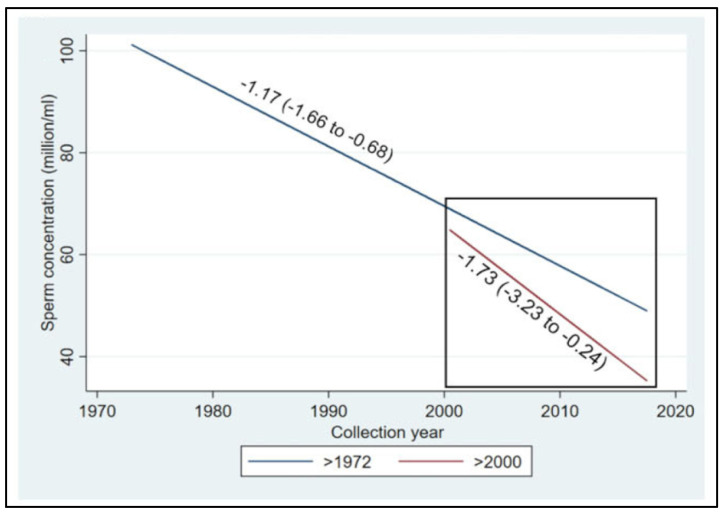
Meta-regression models for mean sperm concentration by collection year among unselected men from all continents, adjusted for potential confounders, for the whole period and additionally restricted to the studies after the year 2000. Sperm concentration decline accelerates from the year 2000 onwards, with a slope of −1.73 million/mL per year as compared with the dynamics of the 20th century, if continued (the blue line in the square). Reprinted from Ref. [4], with permission from Levine, H. et al., Hum. Reprod.; published by Oxford University Press, 2023.

The most recent systematic review and meta-regression analysis of semen samples collected globally in the 20th and 21st centuries confirmed a 50–60% decline in sperm counts among unselected men from all continents, including South and Central America, Asia, and Africa [4]. The 21st century is hallmarked by the acceleration of sperm count decline (Figure 12).

The level of testosterone in adolescent and young adult men as monitored in the USA from 1999 to 2016 was also decreasing by 1% per year [149]. Sperm counts and other semen parameters have been plausibly associated with multiple environmental influences, including endocrine-disrupting chemicals [150,151], pesticides [152], heat [153], lifestyle and diet [154,155], stress [156,157], smoking [158], and elevated body mass index [159,160]. Therefore, sperm count may sensitively reflect the impacts of the modern environment on male health throughout the life course [161], while severe infertility is a marker of genome instability as such [162].

In addition to the global decline of semen quality, an alarming relationship between decreased semen quality and infertility on the one side, and increased morbidity and mortality on the other side, has been observed. It has been reported that men from infertile couples have approximately two-fold more morbidities as compared to their fertile counterparts [163]. Infertile men are at a higher risk of having diabetes, cardiovascular diseases, auto-immune diseases, rheumatic arthritis, and multiple sclerosis [164,165,166]. Infertile men also have a 1.5-fold higher risk of cancer, and a 2-fold higher testicular cancer risk in particular [167]. Moreover, infertile men with the most severe phenotype—azoospermia (a complete absence of spermatozoa in ejaculate) exhibit a 3-fold higher risk of any type of cancer, as compared to men without fertility problems [164].

**Figure 13:**
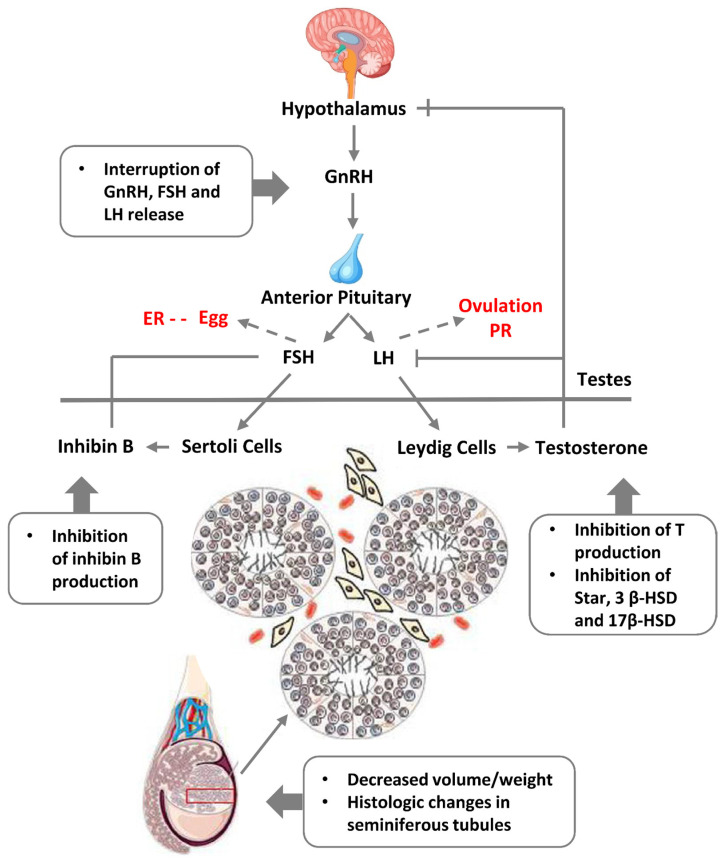
A scheme of the hypothalamic-pituitary-gonadal axis regulating spermatogenesis and the effects of endocrine disruption causing “testicular dysgenesis syndrome”. Abbreviations: GnRH—gonadotropin-releasing hormone; FSH—follicle stimulating hormone; LH—luteinising hormone; ER—oestrogen receptor; PR—progesterone receptor; T—testosteron. Adapted from Ref. [171], CC 3.0 Licence. Modified here with the addition of female regulation pathways (in red font).

Also, it has been shown that decreased sperm quality has a significant correlation with increased mortality [168,169]. More than 20 years ago a Danish group led by Skakkebaek introduced “testicular dysgenesis syndrome (TDS)”—an increasingly common developmental disorder with environmental aspects. They suggested that declining semen quality, increasing testicular cancer, undescended testis, and hypospadias share a common pathogenesis and are the features of the same TDS [170]. Experimental and epidemiological studies showed that TDS is a result of disruption of embryonal programming and gonadal development during foetal life by different endocrine disruptors that have polluted our modern environment, and humans are massively exposed to it through food, water, cosmetics, and construction materials, etc. [150,151]. A scheme of the endocrine disruption of male fertility based on somatic sex determination from [171] modified with the addition of female regulation is given in Figure 13.

## 9. Conclusions

**Figure 14:**
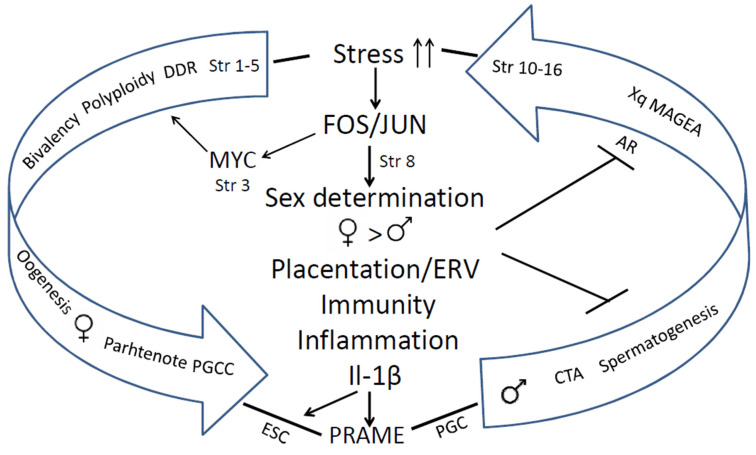
The evolutionary route of the link between reducing male fertility and the increasing risk of cancer emerging in mammals and Eutherian hominids driven by DNA damage and stress response through toggling sex determination and immunity/angiogenesis-invasive (“placentation”) activities towards parthenogenetic cancer. Abbreviations: Str—Phylostrata; DDR—DNA damage response; PGCC—polyploid giant cancer cell; ESC—embryonic stem cell; PGC—primordial germ cell; ERV—endogenous retroviruses.

The human genome gene network is composed of two evolutionary parts—the UC part, encompassing the most essential daily functions which gave birth to the cell cycle, the DNA damage response, meiotic DNA recombinational repair, and gametes. The second is the MC part. The early MC genes are partially ambivalent (Strata 3–5). It can be relatively easy, via the activation of c-Myc, polyploidy, and bivalency, to be united with the dense network of the UC core, providing a basic framework for gametogenesis via cancer pseudo-embryogenetic (parthenogenetic) PGCCs or amoeboid sporogenesis. The main cancer drivers enabling this via mutations or epigenetically are pre-programmed as the normal regulators of the reproductive process that evolved over that same period. Therefore, cancer had already appeared in the *Hydra*. The evolution of UCs and early MCs was very long (~3 Bya) and relatively gradual. The 2R—genome doublings pushing the Cambrian explosion in vertebrates and allowed for the huge variety of newly emerged genes and animals, but the evolution of mammals started only ~250–200 Mya, and that of primates even later, and it had another rate, accelerated by retrotransposon bursts and their DNA insertions in the genomes. It created the incredible complexity of the mammalian and the human brain. The complex biological systems are intrinsically unstable and are thus able to adapt to the environment and change cell fates through exploration and learning [15,172,173]. In this review, we gathered evidence on the features of genome instability and its use in the evolution of mammals and hominids, including segmental genome duplications, increasing retroviral domestication and their ongoing activity, and genome fragility as a source and consequence of both adaptations and cancer. Those also created CTA genes for supporting male reproductivity, counteracting environmental stress with high adaptability (using ERVs for constructing the alternative promoters for other tissues (different from the testis), and intrinsically disordered domains for post-translational switching of cell fate and tissue functions). The reason why only male reproduction needs protection becomes clear from our analysis of the STRING networks in the evolution of reproductive systems: it was created by evolution as female by default, to ensure life continuation in the embryo. We revealed how CTAs also interact with the evolutionary reproduction somatic tools from Euteleostomi, with the shaky for the male somatic sex determination system, and also comply cellular communication presented via the immunity and vascularisation/invasion (“female pregnancy”) system in cancer aggression. It is interesting that we came upon the same crucial significance of immunity for polyploid versus diploid cancers, as Quinton et al. [137] found via differential gene expression analysis through investigating the GG modules, which unmasked the involved proto-placental component of PGCCs. The instability of the human genome and adaptive plasticity in the regulation of CTA genes aiming to stabilise spermatogenesis against environmental stress still appeared a too-fragile instrument in the face of the challenges of environmental pollution, increasing social stress, and particularly endocrine disruption. Both the Scylla of the preferential female gametogenesis-linked carcinogenesis formed at the beginning of UC-MC evolution and the Charybdis of the embryonic male-to-female sex reversal hidden in the sex determination system and established in the vertebrate evolution make the journey of the human male germ differentiation, which is sailed via the CTAs, unsafe, and under stress conditions increases the risk of infertility and gametogenesis-linked cancer, “female” by its origin. The acquisition of the second active X-chromosome through malignant tumours aggravates the story. This story is not contradictory but likely complimentary to the hypothesis of Lavia et al. [174], who proposed a model based on Waddington’s cell differentiation landscape, whereby LINE-1 expression in adult cells triggers chromatin remodelling and reactivates embryonic circuits, ultimately leading to cancer malignancy. The bridge between the two evolutionary branches of human cancer, early and late, arbitrarily “female” and “male”, joined by stress-induced FOS/JUN/MYC immunity- inflammation- linked events, which favours the suppression of male fertility, while increasing the drive towards cancer development (as revealed in our analysis), is schematised in Figure 14.

Without a doubt, many means of combinatorial individual cancer treatments are being and can be further developed, in particular by modulating immunity, which can prolong the life of the patients with metastatic cancers. The most urgent need of mankind for survival is to stop environmental pollution with organic materials, delay atmospheric heating, and shift to a healthier lifestyle.

## Data Availability

Data provided in Table S1.

## Supplementary Materials

The following supporting information can be downloaded at: https://www.mdpi.com/article/10.3390/ijms241411660/s1.
